# Supplementary material for: Fast segmentation with the NextBrain histological atlas
Source: bioRxiv. 2025 Sep 25:2025.09.22.673638. Preprint. [Version 1] doi: 10.1101/2025.09.22.673638 (PMC12485876; doi:10.1101/2025.09.22.673638)
Supplement: Supplement 1 [file NIHPP2025.09.22.673638v1-supplement-1.pdf]

bioRxiv preprint doi: https://doi.org/10.1101/2025.09.22.673638; this version posted September 25, 2025. The copyright holder for this preprint (which was not certified by peer review) is the author/funder, who has granted bioRxiv a license to display the preprint in perpetuity. It is made available under aCC-BY 4.0 International license.

Supplementary Material

Heuristic intensity estimates for creating a synthetic MRI scan

We first compute the median intensities using the coarse segmentation from BrainFM for the white matter (μ^{wm}), gray matter (μ^{gm}), cerebellar white matter (μ^{cwm}), cerebellar gray matter (μ^{cgm}), caudate (μ^{ca}), putamen (μ^{pu}), and pallidum (μ^{pa}). Next the average value and difference between white and gray matter medians is computed:

$$\mu^{\text{mid}} = 0.5(\mu^{\text{wm}} + \mu^{\text{gm}})$$
$$\delta = \frac{\mu^{\text{wm}} - \mu^{\text{gm}}}{16}.$$

The substructure intensities where no direct label correspondence between the coarse BrainFM segmentation and NextBrain exists are created from μ^{mid} and δ as follows:

$\mu^{\text{th-lat}} = \mu^{\text{mid}} + 2\delta$	Lateral part of the thalamus
$\mu^{\text{th-med}} = \mu^{\text{mid}} - 2\delta$	Medial part of the thalamus
$\mu^{\text{rn}} = \mu^{\text{wm}} + 9\delta$	Red nucleus
$\mu^{\text{gm-bs}} = \mu^{\text{wm}} - \delta$	Compact brainstem white matter
$\mu^{\text{wm-bs}} = \mu^{\text{wm}} + 6\delta$	Diffuse brainstem white matter
$\mu^{\text{hypo}} = \mu^{\text{mid}} - 3\delta$	Hypothalamus
$\mu^{\text{ma-body}} = \mu^{\text{wm}}$	Mamillary bodies
$\mu^{\text{dn-cb}} = \mu^{\text{mid}} - \delta$	Dentate nucleus
$\mu^{\text{wm-hippo}} = \mu^{\text{mid}} + \delta$	White matter of the hippocampus

bioRxiv preprint doi: https://doi.org/10.1101/2025.09.22.673638; this version posted September 25, 2025. The copyright holder for this preprint (which was not certified by peer review) is the author/funder, who has granted bioRxiv a license to display the preprint in perpetuity. It is made available under aCC-BY 4.0 International license.

Ex vivo comparison against the manual labeling on (Edlow et al., 2019)

Table S1: The volumes and Dice scores across all ROIs on the *ex vivo* sample from Edlow et al., 2019.

ROI	*Volume mm³*	*Dice*
Whole-cerebral-cortex (all labels combined)	253324	0.89
Cerebral-White-Matter	185615	0.92
Cerebellum-Cortex	41231	0.93
ctx-superiorfrontal	24060	0.75
Cerebellum-White-Matter	17612	0.85
ctx-inferiorparietal	15724	0.79
ctx-precentral	15576	0.76
ctx-rostralmiddlefrontal	15222	0.75
ctx-superiortemporal	13822	0.81
ctx-superiorparietal	13739	0.70
ctx-middletemporal	12339	0.74
ctx-inferiortemporal	11691	0.69
ctx-lateraloccipital	11376	0.57
ctx-postcentral	11098	0.74
ctx-supramarginal	10717	0.76
ctx-precuneus	10375	0.65
ctx-fusiform	9764	0.65
ctx-lateralorbitofrontal	8765	0.76
Brain-Stem	7528	0.54
ctx-insula	7307	0.83
ctx-caudalmiddlefrontal	6708	0.66
ctx-medialorbitofrontal	6172	0.67
ctx-lingual	5552	0.62
ctx-parsopercularis	5507	0.77
Putamen	5132	0.91
ctx-paracentral	4484	0.71
ctx-parstriangularis	4174	0.77
Caudate	3915	0.87
ctx-cuneus	3161	0.42
Pons-corticopontine-pontocerebellar-fibers	2846	0.64
ctx-caudalanteriorcingulate	2788	0.64
ctx-bankssts	2770	0.65
ctx-isthmuscingulate	2704	0.50
ctx-parsorbitalis	2625	0.45
Pons-pontine-nuclei	2549	0.45
ctx-posteriorcingulate	2446	0.53

bioRxiv preprint doi: https://doi.org/10.1101/2025.09.22.673638; this version posted September 25, 2025. The copyright holder for this preprint (which was not certified by peer review) is the author/funder, who has granted bioRxiv a license to display the preprint in perpetuity. It is made available under aCC-BY 4.0 International license.

ROI	*Volume* mm^3	*Dice*
ctx-rostralanteriorcingulate	2362	0.67
ctx-pericalcarine	2258	0.44
ctx-entorhinal	2074	0.64
ctx-temporalpole	2071	0.60
ctx-parahippocampal	1930	0.65
PuL	1559	0.77
external-pallidum	1413	0.78
MDl	1240	0.76
ctx-frontalpole	1028	0.15
ctx-transversetemporal	935	0.61
VA	907	0.71
CA1	890	0.75
Fornix	870	0.29
Claustrum	847	0.53
R	772	0.38
VPL	753	0.64
LP	697	0.65
molecular-layer-HP	673	0.48
internal-pallidum	623	0.74
subiculum	566	0.68
Dentate-cerebellum	563	0.80
alveus	502	0.50
CA4-GC-DG	483	0.70
Accumbens-area	461	0.73
Thalamus	442	0.24
Lateral-nucleus	439	0.78
CeM	393	0.50
Substancia-Nigra	376	0.56
CA2-CA3	271	0.51
AV	254	0.56
Basal-nucleus	253	0.50
SCP	243	0.59
hypothalamus-posterior	234	0.41
hypothalamus-tubular-superior	232	0.53
Accessory-Basal-nucleus	219	0.60
hypothalamus-tubular-inferior	199	0.66
PAG	177	0.59
Red-Nucleus	159	0.91
VTA	157	0.57
STN	145	0.61
Optic-Nerve	142	0.67

bioRxiv preprint doi: https://doi.org/10.1101/2025.09.22.673638; this version posted September 25, 2025. The copyright holder for this preprint (which was not certified by peer review) is the author/funder, who has granted bioRxiv a license to display the preprint in perpetuity. It is made available under aCC-BY 4.0 International license.

ROI	*Volume* mm^3	*Dice*
LGN	124	0.41
acomm	122	0.47
fimbria	111	0.42
MGN	111	0.35
VLa	109	0.34
LD	108	0.23
stria-terminals	106	0.43
Central-nucleus-of-inferior-colliculus	106	0.44
Corticoamygdaloid-transitio	101	0.50
mammillary-body	88	0.64
DR	65	0.25
Inferior-olive	65	0.05
hypothalamus-anterior-superior	57	0.59
Medial-nucleus	49	0.47
Central-nucleus	47	0.35
Anterior-amygdaloid-area-AAA	47	0.15
zona-incerta	41	0.34
hypothalamus-anterior-inferior	32	0.37
Paralaminar-nucleus	27	0.11
Cortical-nucleus	20	0.21
Rest of Hippocampus	<1	N/A
Rest of Amygdala	<1	N/A

bioRxiv preprint doi: https://doi.org/10.1101/2025.09.22.673638; this version posted September 25, 2025. The copyright holder for this preprint (which was not certified by peer review) is the author/funder, who has granted bioRxiv a license to display the preprint in perpetuity. It is made available under aCC-BY 4.0 International license.

Intra-class correlation analysis on the MIRIAD data set

Table S2: The volumes and intra-class correlation coefficients (ICC) over all the ROIs in the NextBrain atlas for the MIRIAD data set. The ROIs are listed in from largest to smallest.

ROI	*Volume mm³*	*ICC*
white-matter-of-forebrain	188896.2	1.00
molecular-layer-of-pva	25508.3	1.00
granular-cell-layer-of-pva	24901.6	1.00
ctx-superiorfrontal	19807.8	0.99
ctx-rostralmiddlefrontal	17267.0	0.99
ctx-precentral	13969.5	0.98
white-matter-of-the-cerebellum	13036.5	0.98
ctx-superiorparietal	12774.2	0.97
ctx-inferiorparietal	12073.0	0.99
ctx-postcentral	11731.9	0.98
ctx-lateraloccipital	11555.3	0.99
ctx-middletemporal	11142.9	0.99
ctx-superiortemporal	11142.1	0.99
ctx-inferiortemporal	10273.6	0.99
ctx-fusiform	10019.9	0.99
ctx-supramarginal	9019.5	0.99
ctx-lateralorbitofrontal	8209.3	0.98
ctx-precuneus	6896.1	0.99
ctx-lingual	6889.9	0.98
ctx-insula	6218.0	0.99
ctx-medialorbitofrontal	5601.3	0.97
ctx-caudalmiddlefrontal	5469.2	0.98
putamen	4293.3	0.99
ctx-parsopercularis	3901.9	0.97
pons	3576.3	0.98
ctx-paracentral	3108.0	0.95
ctx-parsorbitalis	2946.3	0.96
ctx-posteriorcingulate	2738.4	0.96
ctx-cuneus	2677.4	0.98
ctx-parstriangularis	2628.6	0.96
white-matter-of-hindbrain	2509.1	0.98
ctx-isthmuscingulate	2469.0	0.98
ctx-parahippocampal	2432.8	0.99
head-of-caudate	2429.2	0.99
ctx-rostralanteriorcingulate	2401.9	0.92

bioRxiv preprint doi: https://doi.org/10.1101/2025.09.22.673638; this version posted September 25, 2025. The copyright holder for this preprint (which was not certified by peer review) is the author/funder, who has granted bioRxiv a license to display the preprint in perpetuity. It is made available under aCC-BY 4.0 International license.

ROI	*Volume mm³*	*ICC*
ctx-temporalpole	2363.0	0.97
ctx-caudalanteriorcingulate	2296.1	0.91
ctx-pericalcarine	2131.0	0.99
midbrain-(mesencephalon)	2096.2	0.96
ctx-bankssts	2007.3	0.98
ctx-entorhinal	1988.5	0.97
ctx-frontalpole	1707.0	0.94
pontine-nucleus	1173.0	0.97
ctx-transversetemporal	938.4	0.94
external-segment-of-globus-pallidus	937.7	0.98
body-of-caudate	929.3	0.96
myelencephalon-(medulla-oblongata)	927.4	0.89
medial-nucleus-of-pulvinar	612.8	0.99
rostral-subiculum	547.1	0.99
parvocellular-division-of-va	541.3	0.96
dentate-(lateral)-nucleus	514.0	0.93
lateral-posterior-nucleus-of-thalamus	494.2	0.94
claustrum	458.6	0.95
parvocellular-(central)-division-of-md	457.4	0.98
internal-segment-of-globus-pallidus	448.1	0.97
lateral-nucleus	442.7	0.96
thalamus	436.1	0.95
core-of-nucleus-accumbens	391.2	0.97
caudal-division-of-ventral-posterior-lateral-nucleus	353.6	0.95
magnocellular-(medial)-division-of-md	331.9	0.98
stratum-pyramidale-of-rostral-ca1	331.6	0.99
basolateral-nucleus-(basal-nucleus)	328.6	0.99
alveus	296.7	0.97
fornix	258.0	0.96
periaqueductal-gray-substance	250.3	0.93
multiform-(lateral)-division-of-md	248.2	0.96
magnocellular-division-of-va	242.8	0.93
stratum-lacunosum-moleculare-of-rostral-ca1	234.5	0.98
substantia-innominata	230.7	0.95
reticular-nucleus-of-thalamus	225.9	0.94
anterior-nucleus-of-pulvinar	202.2	0.91
superior-cerebellar-peduncle-(brachium-conjunctivum)	186.4	0.95
ventral-division-of-basomedial-nucleus	180.6	0.99
optic-tract	170.3	0.95
lateral-nucleus-of-pulvinar	170.1	0.98
red-nucleus	169.5	0.88

bioRxiv preprint doi: https://doi.org/10.1101/2025.09.22.673638; this version posted September 25, 2025. The copyright holder for this preprint (which was not certified by peer review) is the author/funder, who has granted bioRxiv a license to display the preprint in perpetuity. It is made available under aCC-BY 4.0 International license.

ROI	*Volume mm³*	*ICC*
molecular-layer-of-rostral-dentate-gyrus	164.9	0.99
caudal-subiculum	162.3	0.97
anteroventral-nucleus-of-thalamus	162.1	0.95
dorsal-subdivision-of-vlc	161.1	0.94
rostral-division-of-ventral-posterior-lateral-nucleus	149.2	0.89
substantia-nigra-reticular-part	137.2	0.91
stria-terminalis	132.1	0.91
ventral-tegmental-area	124.1	0.87
rostral-division-of-vl	123.6	0.91
medial-division-of-centromedian-nucleus-of-thalamus	118.7	0.91
bed-nucleus-of-stria-terminalis	118.4	0.94
substantia-nigra–compact-part	117.8	0.91
lateral-dorsal-nucleus-of-thalamus	115.6	0.92
anterior-commissure	112.4	0.97
medial-septal-nucleus	110.5	0.91
amygdalocortical-(corticoamygdaloid)-transition-area	110.3	0.99
temporal-claustrum	109.0	0.90
polyform-layer-of-rostral-dentate-gyrus	108.5	0.98
ventral-subdivision-of-vlc	107.2	0.86
stratum-pyramidale-of-caudal-ca1	101.0	0.97
stratum-pyramidale-of-uncal-ca1	99.1	0.98
anterior-amygdaloid-area	96.8	0.96
central-nucleus-of-inferior-colliculus	93.7	0.91
stratum-radiatum-of-rostral-ca1	92.6	0.98
posterior-nucleus-of-thalamus	87.0	0.92
zona-incerta	78.0	0.92
stratum-lacunosum-moleculare-of-uncal-ca1	77.6	0.98
paraventricular-nucleus	74.8	0.94
densocelllular-(paralamellar)-division-of-md	72.1	0.93
paracentral-nucleus-of-thalamus	71.7	0.93
amygdaloid-complex	70.9	0.98
tail-of-caudate	70.7	0.92
medial-subdivision-of-vlc-(area-x)	70.5	0.83
lateral-division-of-central-lateral-nucleus	67.5	0.89
pyramidal-cells-of-rostral-ca4	65.2	0.99
stratum-oriens-of-rostral-ca1	62.8	0.98
inferior-cerebellar-peduncle	62.8	0.96
supraoptic-region-of-hth	61.7	0.90
lateral-olfactory-area	61.7	0.95
ventral-posterior-inferior-nucleus	59.6	0.93
laterodorsal-portion-of-sth	59.6	0.88

bioRxiv preprint doi: https://doi.org/10.1101/2025.09.22.673638; this version posted September 25, 2025. The copyright holder for this preprint (which was not certified by peer review) is the author/funder, who has granted bioRxiv a license to display the preprint in perpetuity. It is made available under aCC-BY 4.0 International license.

ROI	*Volume mm³*	*ICC*
dorsal-lateral-geniculate-nucleus	59.2	0.93
posterior-hypothalamic-nucleus	57.0	0.86
lateral-hypothalamic-area–tuberal-part	55.1	0.94
dorsal-raphe-nucleus	53.6	0.82
lateroventral-portion-of-sth	52.5	0.85
pretectal-region	52.4	0.92
molecular-layer-of-caudal-dentate-gyrus	51.5	0.97
parafascicular-nucleus-of-thalamus	49.6	0.88
stratum-lacunosum-moleculare-of-caudal-ca1	49.4	0.97
lateral-interpositus-(emboliform)-nucleus	47.4	0.88
lateral-division-of-centromedian-nucleus-of-thalamus	46.6	0.89
mammillothalamic-tract	44.5	0.93
optic-chiasm	41.4	0.87
lateral–hypothalamic-area–posterior-part	41.1	0.89
pallidohypothalamic-area	40.8	0.93
fimbria	40.8	0.97
periventricular-area-of-thalamus	40.4	0.86
inferior-olive–principal-nucleus	38.4	0.88
uncal-subiculum	37.1	0.99
posteroventral-putamen	35.3	0.87
granular-layer-of-rostral-dentate-gyrus	33.1	0.99
inferior-nucleus-of-pulvinar	32.9	0.98
stratum-pyramidale-of-uncal-ca2	32.6	0.98
pulvinar-of-thalamus	31.8	0.97
stratum-pyramidale-of-rostral-ca3	31.8	0.98
ventral-posterior-medial-nucleus	31.2	0.85
reuniens-nucleus-(medioventral-nucleus)-of-thalamus	30.8	0.87
anterior-olfactory-nucleus	30.7	0.92
stratum-pyramidale-of-uncal-ca3	30.2	0.96
tuberomammillary-nucleus	29.2	0.92
central-part-of-medial-division-of-md	28.7	0.79
stratum-radiatum-of-caudal-ca1	28.6	0.97
ventral-pallidus	28.4	0.87
preoptic-region-of-hth	28.3	0.90
stratum-lacunosum-moleculare-of-uncal-ca2	28.0	0.99
stratum-oriens-of-caudal-ca1	27.8	0.96
limitans-nucleus	27.3	0.93
centromedian-nucleus-of-thalamus	27.2	0.93
lateral-preoptic-area	26.5	0.94
polyform-layer-of-caudal-dentate-gyrus	26.0	0.97
medial-interpositus-(globose)-nucleus	25.1	0.91

bioRxiv preprint doi: https://doi.org/10.1101/2025.09.22.673638; this version posted September 25, 2025. The copyright holder for this preprint (which was not certified by peer review) is the author/funder, who has granted bioRxiv a license to display the preprint in perpetuity. It is made available under aCC-BY 4.0 International license.

ROI	*Volume mm³*	*ICC*
stratum-radiatum-of-uncal-ca1	24.5	0.98
ventral-medial-nucleus-of-thalamus	23.3	0.92
rostral-subdivision-of-medial-nucleus	22.4	0.98
molecular-layer-of-uncal-dentate-gyrus	22.4	0.98
amygdalostriatal-transition-area	22.3	0.90
stratum-lacunosum-moleculare-of-rostral-ca3	21.5	0.97
stratum-oriens-of-rostral-ca3	19.8	0.96
medial-subdivision-of-central-nucleus	19.5	0.98
infundibular-stalk	19.5	0.88
medial-portion-of-sth	19.4	0.87
stratum-oriens-of-uncal-ca3	19.4	0.96
stratum-lacunosum-moleculare-of-rostral-ca2	19.2	0.97
amygdalohippocampal-area	18.8	0.99
pyramidal-cells-of-caudal-ca4	18.6	0.97
supraoptic-nucleus	18.3	0.92
arcuate-nucleus-of-hypothalamus	18.1	0.82
mammillary-peduncle	17.4	0.77
anteromedial-nucleus-of-thalamus	17.3	0.84
diffuse-pulvinar-nucleus	17.1	0.97
paraventricular-nucleus-of-hypothalamus	17.0	0.89
magnocellular-division-of-reticular-nucleus	16.8	0.86
diagonal-band	15.3	0.83
parvocellular-division-of-reticular-nucleus-(perireticular-nucleus)	14.9	0.81
inferior-thalamic-peduncle	14.7	0.86
ventral-subdivision-of-coa	14.4	0.98
juxtaparaventricular-lateral-hypothalamic-area	14.4	0.89
basal-nucleus-of-meynert	14.3	0.91
anterodorsal-nucleus-of-thalamus	14.2	0.83
stratum-oriens-of-uncal-ca1	14.0	0.97
stria-medullaris-of-thalamus	13.6	0.89
stratum-lacunosum-moleculare-of-uncal-ca3	13.6	0.97
septum-pellucidum	13.2	0.91
polyform-layer-of-uncal-dentate-gyrus	12.8	0.98
subparafascicular-nucleus-of-thalamus	12.6	0.86
granular-layer-of-caudal-dentate-gyrus	12.6	0.97
fasciculosus-nucleus-of-thalamus	11.9	0.87
parvocellular-division-of-vpm	11.7	0.90
central-part-of-ventromedial-hypothalamic-nucleus	11.3	0.90
lenticular-fasciculus	11.0	0.61
stratum-oriens-of-uncal-ca2	11.0	0.95
medial-part-of-medial-mammillary-nucleus	10.9	0.87

bioRxiv preprint doi: https://doi.org/10.1101/2025.09.22.673638; this version posted September 25, 2025. The copyright holder for this preprint (which was not certified by peer review) is the author/funder, who has granted bioRxiv a license to display the preprint in perpetuity. It is made available under aCC-BY 4.0 International license.

ROI	*Volume mm³*	*ICC*
layer-iii-of-piriform-cortex	10.9	0.92
stratum-pyramidale-of-caudal-ca3	10.7	0.97
stratum-radiatum-of-uncal-ca2	10.5	0.98
stratum-lucidum–of-rostral-ca3	10.4	0.97
dorsal-medial-geniculate-nucleus	10.4	0.89
paralaminar-nucleus	9.8	0.97
lateral-habenular-nucleus	9.7	0.88
stratum-pyramidale-of-rostral-ca2	9.7	0.95
parataenial-nucleus-of-thalamus	9.6	0.84
thalamic-fasciculus	9.5	0.87
dorsal-part-of-ventromedial-hypothalamic-nucleus	9.5	0.92
superficial-pulvinar-nucleus	9.2	0.93
intermediodorsal–nucleus-of-thalamus	9.1	0.80
medial-preoptic-nucleus	9.0	0.92
periventricular-nucleus–tuberal-portion	9.0	0.77
stratum-lucidum–of-uncal-ca3	8.9	0.96
basal-division-of-medial-mammillary-nucleus	8.8	0.81
stratum-lacunosum-moleculare-of-caudal-ca3	8.7	0.96
ventral-part-of-ventromedial-hypothalamic-nucleus	8.7	0.89
amygdalohippocampal-transition-area	8.5	0.98
lateral-olfactory-stria	8.2	0.80
facial-nucleus	8.1	0.86
stratum-lacunosum-moleculare-of-caudal-ca2	7.9	0.98
fastigial-(medial)-nucleus	7.9	0.80
lateral-part-of-medial-mammillary-nucleus	7.5	0.86
periventricular-nucleus–supraoptic-portion	7.3	0.68
central-gray-of-medulla-oblongata	7.1	0.83
stratum-oriens-of-caudal-ca3	7.0	0.94
lateral-tuberal-nuclei	6.8	0.91
anterior-hypothalamic-nucleus	6.6	0.91
stratum-pyramidale-of-caudal-ca2	6.5	0.97
fasciculus-retroflexus-(habenuno-interpeduncular-tract)	6.3	0.79
basal-ventral-medial-nucleus	6.3	0.91
peripeduncular-nucleus	6.0	0.80
lateral-subdivision-of-central-nucleus	5.8	0.98
dorsomedial-hypothalamic-nucleus	5.7	0.91
median-preoptic-nucleus	5.2	0.77
pyramidal-cells-of-uncal-ca4	5.2	0.97
central-medial-nucleus-of-thalamus	4.8	0.70
stratum-radiatum-of-rostral-ca2	4.7	0.89
rhomboid-(central)-nucleus-of-thalamus	4.6	0.78

bioRxiv preprint doi: https://doi.org/10.1101/2025.09.22.673638; this version posted September 25, 2025. The copyright holder for this preprint (which was not certified by peer review) is the author/funder, who has granted bioRxiv a license to display the preprint in perpetuity. It is made available under aCC-BY 4.0 International license.

ROI	*Volume mm³*	*ICC*
stratum-lucidum–of-caudal-ca3	4.0	0.97
nucleus-of-the-field-of-forel	3.9	0.91
granular-layer-of-uncal-dentate-gyrus	3.8	0.97
stratum-radiatum-of-caudal-ca2	3.7	0.97
olfactory-tubercle	3.0	0.87
supraoptic-dicussation	2.8	0.85
stratum-oriens-of-rostral-ca2	2.7	0.94
medial-habenular-nucleus	2.6	0.83
central-dorsal-nucleus-of-thalamus	2.4	0.87
dorsal-division-of-central-lateral-nucleus	2.4	0.94
stratum-oriens-of-caudal-ca2	2.1	0.93
magnocellular-(medial)-nucleus	2.0	0.89
lateral-mammillary-nucleus	1.7	0.64
supramammillary-nucleus	1.2	0.79
optic-nerve	1.0	0.29
suprachiasmatic-nucleus	1.0	0.81
habenular-commissure	0.9	0.86
interstitial-nucleus-of-cajal	0.7	0.88
medial-corticohypothalamic-tract	0.6	0.65
suprageniculate-nucleus-of-thalamus	0.5	0.74
parasubthalamic-nucleus	0.4	0.62
solitary-tract	0.3	0.76
pineal-body	0.2	0.49
